# Dr. Winfield Ayers’ Experience with Mercurol in the Treatment of Syphilis

**Published:** 1902-02

**Authors:** G. Frank Lydston

**Affiliations:** Professor of Genito-Urinary Surgery and Syphilology, State University of Illinois


For Texas Medical Journal.

### Dr. Winfield Ayers' Experience with Mercurol in the Treatment of Syphilis.

BY G. FRANK LYDSTON, M. D.,
Professor of Genito-Urinary Surgery and Syphilology, State University of Illinois.

In the TEXAS MEDICAL JOURNAL (the "Red Back") for January, 1902, appears an abstract of a paper on the treatment of syphilis by Dr. Winfield Ayres, which, I think, should not be allowed to pass without comment. The paper is so manifestly devoted to the exploitation of Mercurol, a supply of which was evidently left at Bellevue hospital for "testimonial" experimentation by the manufacturers, that it possibly should not be taken seriously. However, there are some faults so glaring in the palpably biased report, and the "Red Back" has such an extensive circulation among general practitioners, that I cannot refrain from criticising it. In the first place, I desire to state that the cases are very loosely reported in the abstract, however they may have appeared in the original, which I have not at hand. It does not appear whether the cases were in the initial, the active or the sequelar stage. The opening "opinion" as to the duration of treatment is beyond criticism. It is the "stock" opinion of practically all syphilographers. The statement that the "full dosage" should be given constantly for three years is misleading,—dangerously so. Does the author mean to say that he gives as much mercury as the patient can tolerate for three years without intermission? If so, his syphilitic patients are worthy of remembrance in our prayers. Our author states that in "some cases the protiodide controls the symptoms, but in the majority of cases it is of but little use." The reverse of this is true, as any syphilographer who uses the protiodide understandingly will certify. If Dr. Ayres is serious in his impeachment of the protiodide, I would advise him to try another drug house. Somebody has been imposing upon him. Protiodide of mercury from reliable manufacturers has been found by the majority of spyhilographers to be the most generally useful and most uniformly reliable of all the preparations of mercury for internal use. Dr. Ayres states that the disease was controlled by Mercurol in sixty-four cases of syphilis, as follows: "In two weeks, 8; three weeks, 12; four weeks, 14; five weeks, 6; six weeks, 5; seven weeks, 2; eight weeks, 8; ten weeks, 2; three months, 5, and four months, 1."

The majority of syphilographers will admit that the protiodide frequently shows marked control of active syphilis within one week, and shows a decided curative influence in most cases within two or three weeks. When the lesions begin to retrogress the disease may be said to be under "control." Cases of active early syphilis in which the lesions practically disappear in four to six weeks under the protiodide are so numerous that Dr. Ayres' statements are astonishing. It is usually safe to promise a practical disappearance of the lesions of early active syphilis within six weeks under protiodide, inunctions or injections, the effect of the latter being especially rapid.

And now for his cases:

Case 1 "had been taking bichloride for a month with but little improvement." I am surprised that there was any improvement, and doubt if such as there was could be accredited to the bichloride, which is notoriously unreliable and seldom given save as a tonic or in the form of the mixed treatment in early syphilis by most syphilographers, except by hypodermic injection.

Case 2 "had been under biniodide of mercury and iodide of potassium—which caused iodism." I presume this was an early case. If so, the remedies which had been given were not well selected. In the first place, iodides in early syphilis are chiefly useful as adjuvants to mercury. In the second place, the biniodide, like the bichloride, is notoriously unreliable in syphilis save in the nascent form in the mixed treatment, and even this form is useful in direct ratio to the age of the disease. Granting that the remedies were properly selected, the dosage of the iodide of potassium was too small, and the patient being intolerant of the drug, it could hardly have been pushed to the benefit point in that particular case.

Case 3 "had not been controlled by inunctions of ung. hydrarg. Mercurol was given and the case was under control in seven weeks." If the inunction were not pushed the case is a weak argument. Granting that the ung. hydrarg. was given in sufficient dosage for a sufficient length of time, how did Dr. Ayres differentiate the action of Mercurol from the delayed yet effective action of the inunctions previously given?

Case 4 "had been on three-eights of a grain of hydrarg. biniodide and twenty grains of potassium iodide for two months. This caused nausea and vomiting." The case yielded to Mercurol in three weeks. What sort of an argument is the comparative merit of Mercurol in this case? The biniodide and the potassium iodide were not the proper drugs for early syphilis, and if they had been, the dosage of the biniodide was so large that the object of the treatment was defeated by the resultant gastro-intestinal irritation. The Mercurol acted as any other preparation of mercury might have done, providing it were active and unproductive of gastro-intestinal disturbance.

Case 5 is another case in which Mercurol succeeded in two weeks after bichloride had failed. The same criticisms apply here as in Case 1.

Case 6 had been on "large doses of bichloride for three months, but without effect," and, *mirabile dictu,* without a fatal issue. He was cured by Mercurol in one month. This is really wonderful, considering that as good or better results are often obtained from protiodide, mixed treatment, or tannate of mercury internally, inunctions or injections.

Case 7 received both Mercurol and full doses of potassium iodide. This case is not worth recording in the face of the results obtained from mixed treatment in squamous syphilides. A cure in three weeks under large doses of Mercurol, after a mild course of Mercurol and iodide had been given for some time with marked improvement but no cure, will hardly cause a thrill of excitement in the experienced.

In Case 9 "other forms of mercury had been taken for six months." The author does not state what "forms" nor how they were given. This is the best case of the lot, but, alas, for Mercurol, a period of seven weeks was required for a cure.

In Case 10 the patient "had been taking medicine off and on for two years, but his symptoms had never disappeared entirely." What kind of "medicine" and in what dosage? How much "off" and how much "on"? These points are somewhat important. This is another worthless case. The Mercurol was given in relatively small doses and in combination with potassium iodide.

Mercurol may be of great comparative value in syphilis, but it will require something more than Dr. Ayres' loose and faulty observations to prove its value. Apropos of the question of clinical looseness in general, such reports as Dr. Ayres' are distinctively up-to-date. The country is being flooded with them, and, strange to say, they are usually bolsters for the preparations of some particular manufacturing house. Much of the therapeutic chaos is due to such loosely drawn and prejudiced literature. The author is not always to blame. It has come to pass that our manufacturing houses arrogate unto themselves the assumption that the physician's office is merely an experimental laboratory adjunct to their business. The effrontery with which they pursue the physician with demands that he try this or that preparation with which they flood our offices has assumed somewhat colossal proportions. Even some reliable houses seem to consider the physician as a sort of promoter to exploit their wares, most often without salary, but sometimes—well, sometimes. And this with no reference to the case in point. Dr. Ayres is known to be honest. He may, however, like many another victim of the drug man's wiles, be bored into clinical reports of the action of special preparations. If Mercurol is the best preparation for use in syphilis, let us have it, by all means, but let its merits be established by true scientific methods. It is a remedy of some value, but its superiority to everything else remains to be proved. One thing is certain—it needs to be saved from its friends.

